# Effect of Steel Fibers on the Hysteretic Performance of Concrete Beams with Steel Reinforcement—Tests and Analysis

**DOI:** 10.3390/ma13132923

**Published:** 2020-06-29

**Authors:** Violetta K. Kytinou, Constantin E. Chalioris, Chris G. Karayannis, Anaxagoras Elenas

**Affiliations:** Laboratory of Reinforced Concrete and Seismic Design of Structures, Civil Engineering Department, Faculty of Engineering, Democritus University of Thrace (D.U.Th.), 67100 Xanthi, Greece; vkytinou@civil.duth.gr (V.K.K.); karayan@civil.duth.gr (C.G.K.); elenas@civil.duth.gr (A.E.)

**Keywords:** steel fiber-reinforced concrete (SFRC), cyclic tests, reinforced concrete, direct tension tests, hysteretic response, tension softening, smeared crack model, residual stiffness, finite element (FE) analysis, shear, flexure, numerical analysis

## Abstract

The use of fibers as mass reinforcement to delay cracking and to improve the strength and the post-cracking performance of reinforced concrete (RC) beams has been well documented. However, issues of common engineering practice about the beneficial effect of steel fibers to the seismic resistance of RC structural members in active earthquake zones have not yet been fully clarified. This study presents an experimental and a numerical approach to the aforementioned question. The hysteretic response of slender and deep steel fiber-reinforced concrete (SFRC) beams reinforced with steel reinforcement is investigated through tests of eleven beams subjected to reversal cyclic loading and numerical analysis using 3D finite element (FE) modeling. The experimental program includes flexural and shear-critical SFRC beams with different ratios of steel reinforcing bars (0.55% and 1.0%), closed stirrups (from 0 to 0.5%), and fibers with content from 0.5 to 3% per volume. The developed nonlinear FE numerical simulation considers well-established relationships for the compression and tensional behavior of SFRC that are based on test results. Specifically, a smeared crack model is proposed for the post-cracking behavior of SFRC under tension, which employs the fracture characteristics of the composite material using stress versus crack width curves with tension softening. Axial tension tests of prismatic SFRC specimens are also included in this study to support the experimental project and to verify the proposed model. Comparing the numerical results with the experimental ones it is revealed that the proposed model is efficient and accurately captures the crucial aspects of the response, such as the SFRC tension softening effect, the load versus deformation cyclic envelope and the influence of the fibers on the overall hysteretic performance. The findings of this study also reveal that SFRC beams showed enhanced cyclic behavior in terms of residual stiffness, load-bearing capacity, deformation, energy dissipation ability and cracking performance, maintaining their integrity through the imposed reversal cyclic tests.

## 1. Introduction

Short discrete fibers are used as mass reinforcement in concrete structural members to enhance tensile characteristics and to control crack width by the crack-bridging phenomenon observed at a local crack. The structural advances of fiber-reinforced cement-based members depend mainly on the content and the geometrical and mechanical properties of the fibers. Each fiber type influences in some particular function acting as crack arrestors due to their strength, bond, and pullout mechanisms across a crack surface. The addition of fibers to concrete has been shown to increase its toughness significantly and to promote more ductile material behavior [1,2,3,4,5,6]. The incorporation of randomly distributed short steel fibers has been developed as an alternative reinforcement technique to improve the brittle tensional failure and poor cracking performance of concrete by the debonding and pullout process of the fibers [7,8,9]. For this reason, deformed steel fibers with hooked ends exhibit anchoring action, providing increased bond characteristics that leads to a crucial enhancement of the post-cracking response, improving the energy-absorbing capability [10,11,12].

Reinforced concrete (RC) structural members with a low shear span-to-depth ratio, such as deep beams, are shear-vulnerable exhibiting brittle failure due to the low tensile properties of concrete [13]. The favorable influence of fiber-reinforced concrete inspired researchers to study the application of fibers in shear-critical RC beams instead of conventional transverse steel reinforcement [14,15,16]. In beams, the addition of steel fibers improves the concrete’s diagonal tension capacity, leading to increased shear resistance, which can promote flexural failure and ductility [17,18]. Although the full replacement of conventional steel stirrups with fibers proved to be rather difficult, at least a partial replacement of closed stirrups with steel fibers leading to desirable reduce of conventional reinforcement congestion could be possible under certain circumstances [19,20,21]. The replacement of steel stirrups is important in shear-deficient RC joints [22,23,24,25], columns [26,27], deep [28] and torsional beams [29], where design criteria usually require a high amount of transverse reinforcement leading to dense placing of stirrups or the installation of cumbersome reinforcing systems. In this direction, a significant contribution is the recent analytical models that have been proposed to predict the shear capacity of steel fiber-reinforced concrete (SFRC) beam with [30,31] or without stirrups [32,33,34].

Reinforced concrete is capable of bearing some tensile stresses between cracks; this effect is called tension stiffening and is responsible for increased tensile stiffness in the RC member before the yielding of reinforcement [35]. Rigidity, deflections and crack widths may be greatly affected by this effect under the service limit state. The inclusion of steel fibers in the concrete matrix can reduce control crack splitting and significantly enhances residual stiffness, as SFRC can bear tensile stresses along the cracks [36,37,38,39,40]. However, the very restricted usage of SFRC in structural applications is mostly related to the difficulties in determining accurate and logical approaches that represent and estimate the performance of the material in either service or ultimate limit state. At flexure ultimate limit state, the tensile strength of SFRC members is usually disregarded [41,42]. Nevertheless, the member is stiffer as SFRC can bear tensile stresses both across cracks and among them. Thus, as strain increases and cracks coalesce, adequate residual tensile stresses are still developed since SFRC utilizes the fiber crack bridging effect, the tension stiffening attributed to steel reinforcement bond with concrete and the fracture mechanics of SFRC. This residual stiffness must be included in the calculations for the design of SFRC structural members, as it has been observed that the residual stresses get increased by adding higher amounts of steel fibers in the concrete mixture [43,44].

It is known that steel fibers and conventional steel reinforcing bars with stirrups are usually combined. In such cases, the tensile stresses that are being developed at a crack are distributed to the reinforcing bars and steel fibers bridging the crack. This way, the added steel fibers enhance the residual stiffness, provide crack control and enables the usage of higher strength steel reinforcement while retaining the control of crack widths based on the type and amount of steel fibers added [45,46]. These effects have become vital in determining cracking processes at service loads and in developing accurate constitutive models of cracked SFRC, which can be used in an analysis to predict member behavior [47,48].

It is recognized that the effective design of SFRC structures against complex external actions, such as earthquake, fatigue, explosion, and other types of loads, require a clear understanding of SFRC’s mechanical behavior under monotonic and cyclic loads. Cyclic loading is one of the most complex loading conditions, both in structural performance and from a modeling point of view. Researchers have studied the fatigue life of fiber-reinforced high-strength concretes to determine the number of cycles that the specimen can withstand [49,50]. Others have studied cyclic tensile behavior of SFRC to reveal the underlying damage mechanism and SFRC specimens subjected to cyclic compressive load to a variety of high-stress range and high strain rate [51,52,53]. There are only a few cyclic experimental tests on SFRC beams under flexure [54,55,56,57,58,59]. Besides, while there is extensive research on the shear behavior of SFRC subject to monotonic loading, there is minimal research on the behavior of shear-critical SFRC structural members subject to reverse cyclic loading [60,61,62].

From the above literature review, it can be summarized that although some attempts have been made to capture the cyclic response of SFRC, broad experimental studies on the reverse cyclic behavior of SFRC have still been limited. The cyclic stress–strain relation and the damage evolution law of SFRC materials are some of the critical aspects in uncovering the realistic failure process as well as probing the variation of the structural responses of SFRC structures.

The present study aims to contribute to the ongoing research on SFRC by examining the significance of considering the tension softening and residual stiffness effect in SFRC beams subjected to reverse cyclic loading failing in flexure or shear. The precise definition of how this effect progresses with the number of cycles has not yet been adequately investigated as far as SFRC is concerned. For this purpose, a research program has been carried out herein, combining experimental investigation and numerical modeling. Precise finite element (FE) simulation that has been established with test data enables to explore even more the parameters affecting and to solve difficult structural problems [63,64,65].

The experimental part of this study includes two series of concrete beams with conventional steel reinforcing bars, stirrups, and short steel fibers. The first series consists of two slender SFRC beams tested under displacement control cyclic loading conducted by Chalioris et al. [66]. The second series includes nine deep SFRC beams subjected to a force control cyclic reversal loading for the purposes of this study. This test project brings new data concerning the improvement of the hysteretic behavior of realistic beams under reversal loading due to the addition of steel fibers. That might broaden the application of SFRC to structures in regions with high seismic activity.

Furthermore, a computationally efficient simulation using ABAQUS [67] software that takes into account the nonlinearities of the SFRC is also presented. The developed nonlinear FE analysis allows for an accurate prediction of the overall hysteretic response of realistic SFRC members. Comparisons between test and numerical results showed the feasibility of the proposed approach to simulate the response of flexural and shear-critical SFRC beams subjected to cyclic deformations. The effect of steel fibers on the overall performance and cracking behavior is also presented and discussed.

## 2. Materials and Methods

### 2.1. Experimental Investigation

The experimental part of this study includes two series of concrete beams with conventional steel reinforcing bars, stirrups, and short steel fibers. The first series consists of two slender SFRC beams tested under displacement control cyclic loading that was conducted by Chalioris et al. [66]. The second series includes nine deep SFRC beams subjected to a force control cyclic reversal loading for the purposes of this study. Details of the beam specimens are presented in this section.

#### 2.1.1. Characteristics of the Beam Specimens

Beams are sorted in three groups (“FL”, “SH-s” and “SH”) as shown in Table 1 and described below:

Group “FL” includes the two slender beams (2500 mm long) failed in flexure (specimens FL0.3 and FL1.0) containing 1% and 3% steel fibers per volume fraction, *V_SF_*, respectively, that correspond to 80 kg and 240 kg per cubic meter of concrete, respectively. The added steel fibers are hooked-ended with a length to diameter ratio (aspect ratio) of *l_SF_/d_SF_* = 44 mm/1 mm = 44, a bond factor of *β* = 0.75 and, therefore, the fiber factor of beams FL0.3 and FL1.0 is *F* = 0.3 and 1.0, respectively. The geometrical and the reinforcement characteristics of these flexural beams are presented in Table 1 and further details can also be found in the recent study of Chalioris et al. [66].

All deep beams (nine shear-critical specimens) are 1600 mm long, have the same width to height ratio *b*/*h* = 100/300 mm, effective depth *d* = 275 mm, shear span *a_s_* = 550 mm, shear span to effective depth ratio *a_s_*/*d* = 2 and three longitudinal steel reinforcing bars with a diameter of 8 mm at the top and at the bottom with geometrical longitudinal reinforcement ratio *ρ_l_*_1_ = *ρ_l_*_2_ = 0.55% and *f_y_* = 575 MPa. Deep beams are sorted in two groups “SH-s” and “SH”, with and without closed steel stirrups, respectively.

Group “SH-s” includes four deep beams (specimens SH0-s37, SH0-s50, SH0.3-s37 and SH0.3-s50) with stirrups of 8 mm diameter at a uniform spacing of 275 and 200 mm that corresponds to a geometrical web (transverse) reinforcement ratio of *ρ_w_* = 0.37% (specimens SH0-s37 and SH0.3-s37) and *ρ_w_* = 0.50% (specimens SH0-s50 and SH0.3-s50), respectively. Beams SH0-s37 and SH0-s50 are made of plain concrete (reference specimens), whereas beams SH0.3-s37 and SH0.3-s50 contain steel fibers with *V_SF_* = 0.5%.

Group “SH” consists of five deep beams without stirrups (specimens SH0, SH0.3, SH0.4, SH0.6 and SH0.8) that contain steel fibers with *V_SF_* = 0 (plain concrete reference beam), 0.5%, 0.75%, 1.0% and 1.5%, respectively.

**Figure 1:**
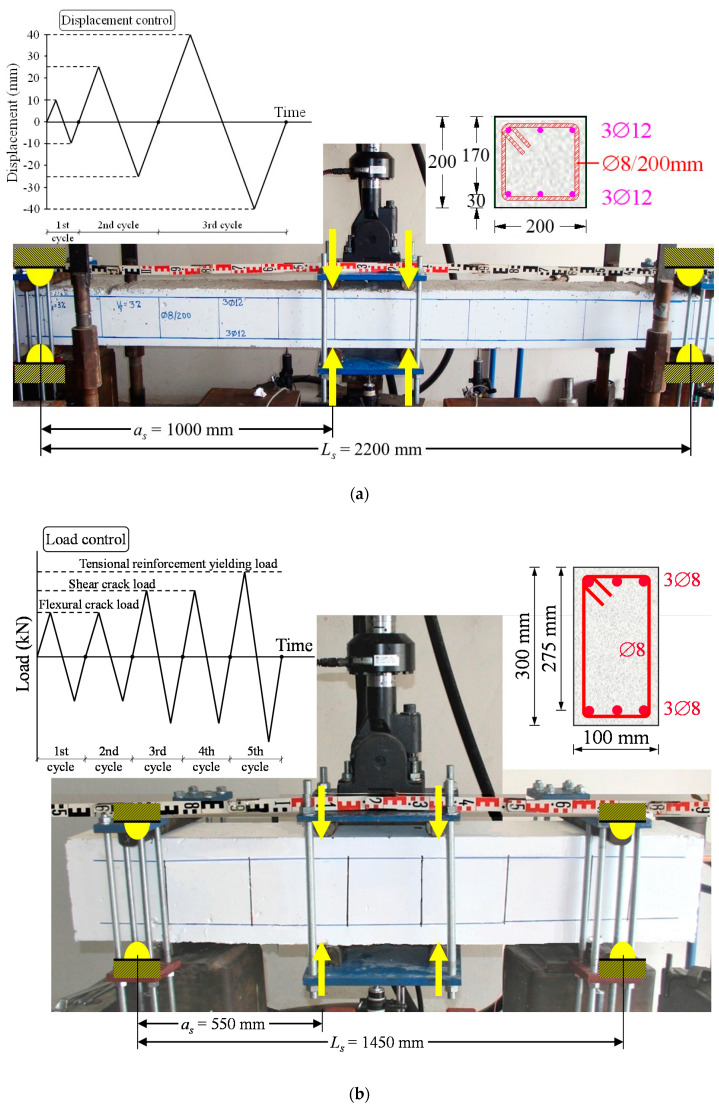
Geometry, reinforcement and cyclic reversal loading sequence of the tested specimens (dimensions in mm): (**a**) slender (flexural) beams; (**b**) deep (shear-critical) beams.

The steel fibers added in the deep beams are hooked ended with a length to diameter ratio (aspect ratio) of *l_SF_/d_SF_* = 60 mm/0.8 mm = 75, bond factor *β* = 0.75 and, therefore, the fiber factor of the SFRC beams of group “SH-s” is *F* = 0.3 and of group “SH” is *F* = 0.3, 0.4, 0.6 and 0.8, as shown in Table 1. Geometrical and reinforcement characteristics of all the examined beams are also summarized in Table 1 and illustrated in Figure 1. Details concerning the preparation stages, the mixture procedure and the curing of steel fiber reinforced concrete specimens can be found in [66].

#### 2.1.2. Test Rig and Loading Histories

Beams were imposed to full-cycle deformations and subsequently to increasing load till failure. The test setup is shown in Figure 1a,b for the slender and the deep beams, respectively. A four-point-bending test setup was implemented for the cyclic reversal loading of the beams that were simply edge-supported on roller supports 2200 mm (slender beams) and 1450 mm (deep beams) apart in a rigid laboratory frame.

The imposed load was applied in two points in the mid-span of the beams and therefore the shear span, *a_s_*, is equal to 1000 mm (slender beams) and 550 mm (deep beams) with span-to-depth ratio: *a_s_*/*d* = 5.9 (slender beams) and 2.0 (deep beams). The load was measured by a load cell with 0.05 kN accuracy and net midspan deformations by LVDTs with 0.01 mm accuracy that were placed in the midspan of the beams and the supports. The load and corresponding deflection measurements were recorded continuously during the performed cyclic tests.

Beams of group “FL” (flexural specimens) were tested under increasing cyclic loading history that includes three loading steps as shown in Figure 1a. The loading history of the deep beams of group “SH” (specimens without stirrups) and the beams of group “SH-s” (specimens with stirrups) includes three and five full loading cycles, respectively, as shown in Figure 1b. The first two loading cycles correspond to the load for the initiation of the flexural crack, the next two to the load for the onset of the inclined shear cracking and the last one almost to the load for the steel yielding of the tension bars of the reference beams SH0, SH0-s37 and SH0-s50 (specimens without steel fibers).

#### 2.1.3. Properties of the SFRC

The plain concrete and SFRC mixture used consisted of an ordinary general-purpose Portland-type cement (type CEM II 32.5 N, Greek type pozzolan cement with 10% fly ash), high fineness modulus sand, crushed stone aggregates with a maximum size of 16 mm (for the slender beams of group “FL”) and 9.5 mm (for the deep beams of groups “SH-s” and “SH”) and a water-to-cement ratio of 0.55 and 0.57, respectively. Uniaxial compression and tension tests were also performed to measure the compressive strength and the full tensile behavioral curves of the SFRC mixtures at the day of the cyclic tests of the beams (after 28 days of curing).

Three standard concrete cylinders with dimensions diameter to height = 150/300 mm were cast from each plain concrete and SFRC mixture and tested under axial compression using a universal testing machine (UTM) with an ultimate capacity of 3000 kN. The average compressive strength of the concrete without steel fibers is 27 MPa, whereas the average compressive strength, *f_c,SF_*, of each SFRC beam is presented in Table 2.

**Figure 2:**
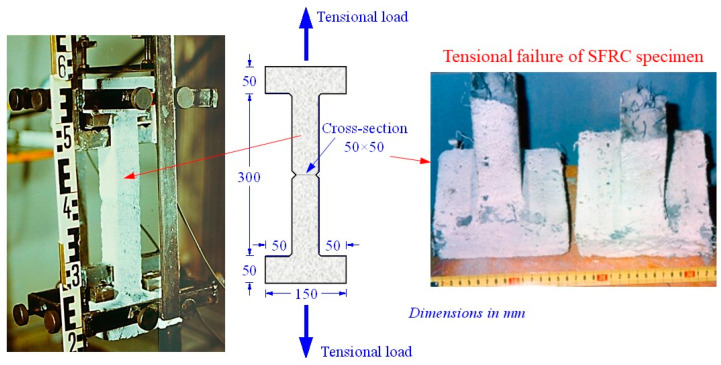
Prismatic SFRC specimen tested and failed under axial tension test.

Concerning the tensile behavior of SFRC, three prismatic specimens were cast from each SFRC mixture and tested under axial tension. Figure 2 illustrates the geometry of the specimens and the direct tension test setup. The extension rate was 0.02 mm/m/sec and tensile deformations and crack width were monitored through four LVDTs with 0.001 mm accuracy that were placed symmetrically on two steel hoops fixed on the wide edges of the specimen. It is noted that tensile cracking and final failure occurred in the middle of the gauge length area of the SFRC notched specimens, away from the edge-mounted clamps, as shown in Figure 2.

**Figure 3:**
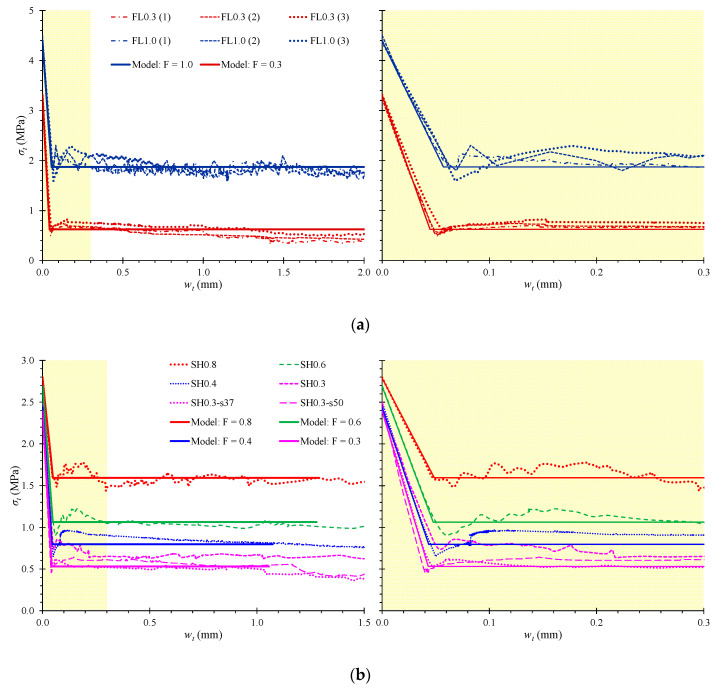
Tensional stress versus crack width (*σ_t_* - *w_t_*) behavioral curves obtained from axial direct tension tests and predicted from the proposed model: (**a**) SFRC mixtures of the slender beams (three tensional specimens from each SFRC mixture); (**b**) SFRC mixtures of the deep beams.

The experimentally measured stress versus crack width behavior of each SFRC mixture under direct tension are presented in Figure 3a,b for the slender and the deep beams, respectively. For comparison reasons, the predictions of the proposed smeared crack tensional model in stress versus crack width curves for each SFRC beam are also illustrated in Figure 3a,b and compared the test results. The initial tensile behavior before cracking was elastic and linear to the point of the SFRC tensile strength, *f_t,SF_*, with elastic modulus under tension, *E_t,SF_* (see also Table 2). The main SFRC variables derived from the tests and the proposed model are also presented in Table 2 for each beam.

The experimental results of the direct tension tests indicate that steel fibers substantially influence the tensile behavior after cracking. The descending post-peak part of the tensile response, especially the residual stress versus crack width curve, depends on the fiber factor, *F*, of the fibers added in the mixtures. The test curves indicate that SFRC with a higher content of steel fibers (see for example beams FL1.0 and SH0.8 with *V_SF_* = 3% and 1.5%, respectively, and *F* = 1.0 and 0.8, respectively) demonstrate higher post-cracking stress with regard to the corresponding SFRC mixtures with lower steel fiber content (see for example beams FL0.3 and SH0.3 with *V_SF_* = 1% and 0.5%, respectively and *F* = 0.3, both of them).

It is also deduced that the fiber factor, *F*, is a more efficient parameter than the volume fraction, *V_SF_*, for the evaluation of the steel fiber contribution on the residual stress since SFRC mixtures with the same fiber factor *F* = 0.3 and different steel fiber content, such as beam FL0.3 with *V_SF_* = 1% and beams SH0.3, SH0.3-s37 and SH0.3-s50 with *V_SF_* = 0.5% exhibit more or less the same post-cracking stress (approximately 0.6 MPa), as shown in Figure 3a,b.

Furthermore, the comparison between the experimental and the analytical diagrams of Figure 3a,b reveals that the stress versus crack width curves derived from the proposed smeared crack analysis that takes into account the tension softening phenomenon are in very good compliance with the test results.

### 2.2. Proposed Model, Constitutive Relationships of the Materials and Nonlinear FE Analysis

A nonlinear 3D FE analysis has been performed to predict the response of slender and deep SFRC beams with steel reinforcement subjected to reversal cyclic loading. This way, the efficacy and accuracy of the developed FE model are checked using experimental data of SFRC beams failing in flexure and in shear. The performed analysis adopts properly modified constitutive laws of the materials that consider the influence of the added steel fibers to the compressive and to the tensile behavior of SFRC with tension softening and residual stiffness effect.

The constitutive laws of SFRC under reversal compression and tension are based on test results and models that have been addressed by the authors in previous relative studies. Especially for the simulation of the tensional response of SFRC, a smeared crack analysis with tension softening is adopted. This model uses the fracture characteristics of the material taking into account stress versus crack width constitutive laws with post-cracking descending part (softened tensional behavior). The post-cracking response of SFRC under tension near the reinforcing bars is simulated by a residual stiffness approach that combines the interaction of steel fibers in the cracked concrete regions, the bond performance of steel reinforcing bars corresponding to the tension stiffening effect, the reinforcement characteristics and the fracture mechanics of SFRC. Furthermore, the performed FE analysis utilizes the concrete damaged plasticity (CDP) approach [68] to simulate the behavior of SFRC.

#### 2.2.1. SFRC under Reversal Compression

It is known that steel fibers become more effective after cracking and, therefore, they mainly improve the post-peak compressive response of SFRC elements. The content, the geometrical and the bond properties of the added fibers are the main parameters that influence the enhanced post-cracking behavior of SFRC under compression [69,70,71]. The ultimate stress is slightly increased due to the presence of steel fibers since their progressive debonding failure improves the crack growth resistance of the material that undergoes after the compressive strength. Most of the proposed models of the literature that simulate the SFRC under compression are based on test results and proper regression analysis [72,73,74,75,76].

**Figure 4:**
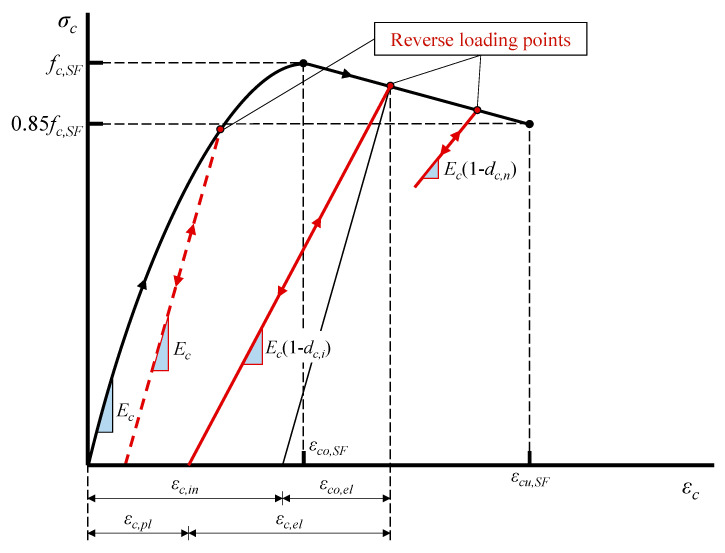
Model of SFRC under reversal compression.

The proposed model for SFRC under reversal compression is illustrated in Figure 4. It can simulate the observed properties of the material behavior, such as the cracking and crushing of SFRC, the accumulation of damage (fracture), and the degradation of stiffness under cyclic loading. The SFRC constitutive law can be defined using multiple points on the compressive stress-strain (*σ_c_* - *ε_c_*). A user-defined damage curve is implemented in the proposed FE analysis to account for the gradual SFRC stiffness degradation as the cracks spread. When the unloading takes place after the first crack formulation, the unloading branch stiffness is equivalent to the elastic stiffness, decreased by a factor, *d_c_*, which considers the degradation due to damage. A substantial decrease in the stiffness is observed up to the full closure of the crack. The crack closure stage ends when the elastic displacement is restored. At this point, the elastic stiffness is also restored, and tension is performed.

The uniaxial compressive behavior of SFRC in relation to the CDP model can be formulated using stress versus plastic strain curves. The stress and the strain parameters of the proposed reversal compressive response shown in Figure 4 are calculated as follows:

(1) $$\sigma_{c}=\left(1-d_{c}\right)E_{c}\left(\varepsilon_{c}-\varepsilon_{c,pl}\right),$$

(2) $$\varepsilon_{c,pl}=\varepsilon_{c,in}-\frac{d_{c}}{1-d_{c}}\varepsilon_{co,el},$$

(3) $$\varepsilon_{c,in}=\varepsilon_{c}-\varepsilon_{co,el},$$

(4) $$\varepsilon_{co,el}=\sigma_{c}/E_{c},$$

where *E_c_* is the initial elastic modulus of SFRC under compression and *d_c_* is the compressive plastic damage factor that takes values 0 ≤ *d_c_* ≤ 1: 0 for the undamaged SFRC and 1 for the complete loss of the SFRC compressive strength, *f_c,SF_*:

(5) $$d_{c}=1-\sigma_{c}/f_{c,SF},$$

The analytical formulation of the compressive stress–strain behavior of SFRC adopted in this study has been derived from test data of 125 stress versus strain curves and 257 strength values [77]. The ascending part of the compressive behavior until the ultimate strength of commonly used SFRC with *f_c,SF_* ≤ 50 MPa is expressed by (see also Figure 4):

(6) $$\sigma_{c}=f_{c,SF}\left[1-{\left(1-\frac{\varepsilon_{c}}{\varepsilon_{co,SF}}\right)}^{2}\right],$$

where *ε_co,SF_* is the strain corresponding to the maximum compressive stress, *f_c,SF_*, of the SFRC.

Concerning the post-peak compressive behavior, a linear descending part is considered from the ultimate strength, *f_c,SF_*, until the value of 0.85*f_c,SF_* is obtained. The SFRC compressive strength, *f_c,SF_*, the corresponding strain, *ε_co,SF_*, and the strain, *ε_cu,SF_*, corresponding to the stress value 0.85*f_c,SF_* are estimated by [77]:

(7) $$f_{c,SF}=f_{c}\left(0.2315F+1\right),$$

(8) $$\varepsilon_{co,SF}=\varepsilon_{co}\left(0.95F+1\right),$$

(9) $$\varepsilon_{cu,SF}=\varepsilon_{co,SF}\left(1.40F+1\right),$$

where *f_c_* is the compressive strength of plain concrete, *ε_co_* is the corresponding strain that is usually equal to 0.002, *F* is the fiber factor $F=\beta V_{SF}\left(l_{SF}/d_{SF}\right)$, where *β* is a bond factor (taken 0.50 for round, 0.75 for deformed and 1.0 for indented fibers), *V_SF_* is the volume fraction of the steel fibers and *l_SF_* and *d_SF_* are their length and diameter, respectively.

#### 2.2.2. SFRC under Reversal Tension

**Figure 5:**
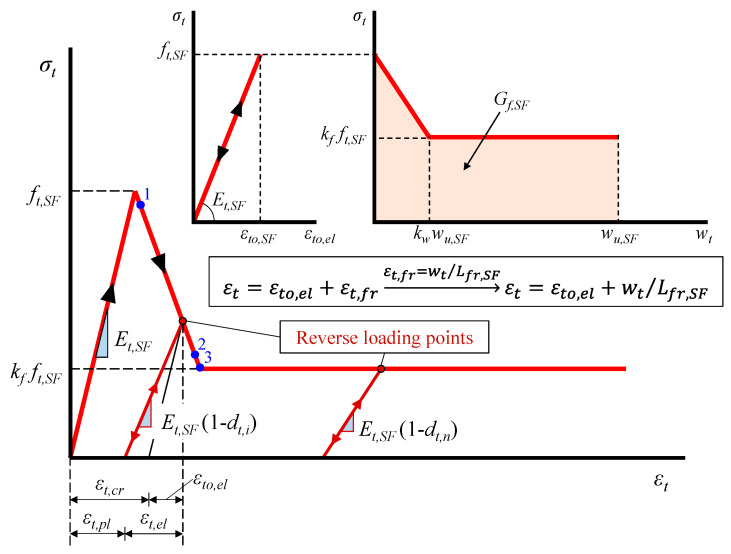
Model of SFRC under reversal tension.

The performance of concrete under tension can be substantially improved by the addition of steel fibers since SFRC exhibits increased tensile strength and mainly post-peak deformation capability showing pseudo-ductile behavior due to the gradual debonding failure of the fibers [78]. Various analytical stress versus strain expressions have been proposed to simulate the SFRC tensile behavior [79,80,81]. In this study, a smeared crack model for plain concrete with tension softening that has been addressed and experimentally verified by the authors [82,83] is adopted. This model has properly been modified to simulate the favorable influence of steel fibers in SFRC under tension. Smeared crack approaches have also been used to evaluate the uncertainty of crack width in large-scale RC beams [84]. Furthermore, the uniaxial tensile response of SFRC in relation to the CDP model can be formulated using stress versus plastic strain curves. The parameters used in the proposed reversal tensile behavior shown in Figure 5 are calculated as follows:

(10) $$\sigma_{t}=\left(1-d_{t}\right)E_{t,SF}\left(\varepsilon_{t}-\varepsilon_{t,pl}\right),$$

(11) $$\varepsilon_{t,pl}=\varepsilon_{t,cr}-\frac{d_{t}}{1-d_{t}}\varepsilon_{to,el},$$

(12) $$\varepsilon_{t,cr}=\varepsilon_{t}-\varepsilon_{to,el},$$

(13) $$\varepsilon_{to,el}=\sigma_{t}/E_{t,SF}\;,$$

where *E_t,SF_* is the initial elastic modulus of SFRC under tension and *d_t_* is the tensile damage factor, which takes values 0 ≤ *d_t_* ≤ 1: 0 for the undamaged SFRC and 1 for the complete loss of the SFRC tensile strength, *f_t,SF_*:

(14) $$d_{t}=1-\sigma_{t}/f_{t,SF},$$

The analytical formulation of the proposed smeared crack approach for the post-cracking tensile behavior of SFRC utilizes stress versus crack width relationships. SFRC cracking takes place within a fracture process zone that is initiated at the tensile strength of SFRC, *f_t,SF_*. The boundary of the strain-softening region and the SFRC characteristics define this zone, assuming that less damaged or even elastic parts coexist between the cracks of this fracture process zone. The total tensional strain, *ε_t_*, is estimated as the sum of an elastic, *ε_to,el_*, and a fracture component, *ε_t,fr_* (see also Figure 5):

(15) $$\varepsilon_{t}=\varepsilon_{to,el}+\varepsilon_{t,fr},$$

(16) $$\varepsilon_{t,fr}=w_{t}/L_{fr,SF},$$

where *σ_t_* is the tensile stress, *w_t_* is the crack width and *L_fr,SF_* is the fracture process zone length that can be taken equal to 3*l_sf_* [85].

The following equations describe the elastic component of the model (see also the linear *σ_t_* - *ε_to,el_* diagram of Figure 5) [86]:

(17) $$f_{t,SF}=\varepsilon_{to,SF}E_{t,SF},$$

(18) $$\varepsilon_{to,SF}=0.167n_{l,el}V_{SF}\left(f_{SF}/E_{SF}-f_{t}/E_{t}\right)+f_{t}/E_{t},$$

(19) $$E_{t,SF}=\frac{3}{8}\left[E_{t}\left(1-V_{SF}\right)+E_{SF}V_{SF}\right]+\frac{5}{8}\left[\frac{E_{SF}E_{t}}{E_{SF}\left(1-V_{SF}\right)+E_{t}V_{SF}}\right],$$

where *E_t,SF_* is the modulus of elasticity under tension of SFRC, *f_t_* and *E_t_* are the ultimate tensile strength and the elastic modulus under tension of the plain concrete, respectively, *f_SF_* and *E_SF_* are the tensile strength and the elastic modulus of the steel fiber, respectively, and *n_l,el_* is the ratio of the average elastic stress to the strength of the steel fiber that is usually equal to 0.5 [86].

The properties of the SFRC softening and fracture response define the parameters of the fracture components of the proposed smeared crack approach [87,88]. The fracture energy, *G_f,SF_*, is the energy required for the cracking formation within the fracture process zone and for the full opening of one single crack [89]:

(20) $$G_{f,SF}=\int_{f_{t,SF}}^{0}\sigma_{t}dw_{t}\overset{w_{t}=L_{fr,SF}\varepsilon_{t,fr}}{\to }G_{f,SF}=L_{fr,SF}\int_{f_{t,SF}}^{0}\sigma_{t}d\varepsilon_{t,fr},$$

The post-peak behavior shown in the *σ_t_* - *w_t_* curve of Figure 5 is defined by a linear descending part until the point of the maximum post-cracking tensional stress, *k_f_ f_t,SF_*, and the corresponding crack width, *k_w_w_u,SF_*. After this point, the stress is assumed to have the following constant value until the ultimate crack width, *w_u,SF_*: *σ_t_* = *k_t_ f_t,SF_* (*w_t_* > *k_w_w_u,SF_*). The fracture energy is the area under the curve of SFRC stress versus crack width (see also the bilinear *σ_t_* - *w_t_* diagram of Figure 5):

(21) $$G_{f,SF}=f_{t,SF}w_{u,SF}\left(k_{f}+0.5k_{w}-0.5k_{f}k_{w}\right),$$

where the values of the coefficients *k_f_* and *k_w_* depend on the SFRC characteristics as [85]:

(22) $$k_{f}=\frac{0.405n_{l}\sigma_{fu}V_{SF}}{\varepsilon_{to,SF}E_{t,SF}},$$

(23) $$k_{w}=\frac{\left(3÷8\right)L_{fr,SF}\varepsilon_{to,SF}}{w_{u,SF}}$$

where *σ_fu_* is the maximum stress of the fiber when a uniform bond stress, *τ_u_*, is assumed at the interface between the steel fiber and the concrete:

(24) $$\sigma_{fu}=\left\{\begin{array}{cc} 2\tau_{u}l_{SF}/d_{SF} & l_{SF}\leq l_{cr}\\ f_{SF} & l_{SF}>l_{cr} \end{array}\right\},$$

(25) $$n_{l}=\left\{\begin{array}{cc} 0.50 & l_{SF}\leq l_{cr}\\ 1-\frac{l_{SF}}{2l_{cr}} & l_{SF}>l_{cr} \end{array}\right\},$$

where *l_cr_* is the length of the fiber in which the ultimate fiber stress is developed:

(26) $$l_{cr}=0.5f_{SF}d_{SF}/\tau_{u},$$

The fracture energy, *G_f,SF_*, can been estimated by tension tests as a function of the known steel fiber factor, *F*, and the value of the fracture energy of the plain concrete, *G_f_* [85]:

(27) $$G_{f,SF}=G_{f}\left(104F+1\right),$$

where the fracture energy of the plain concrete, *G_f_*, can be calculated using linear relation from the tensile strength of concrete, *f_t_*, to zero at the maximum crack width, *w_u_*:

(28) $$G_{f}=0.5f_{t}w_{u},$$

(29) $$w_{u}=\varepsilon_{tu,fr}L_{fr}\overset{\varepsilon_{tu,fr}=a_{fr}\varepsilon_{to}}{\to }w_{u}=a_{fr}\varepsilon_{to}L_{fr}\overset{\varepsilon_{to}=f_{t}/E_{t}}{\to }w_{u}=a_{fr}L_{fr}\frac{f_{t}}{E_{t}}$$

where *a_fr_* is a coefficient that takes values from 5 to 8 for maximum aggregate size *d_g_* = 32 to 8 mm, respectively [82], and *L_fr_* is the plain concrete fracture process that can be taken as 3*d_g_* [90]). This way, Equation (21) can be written as:

(30) $$f_{t,SF}w_{u,SF}\left(k_{f}+0.5k_{w}-0.5k_{f}k_{w}\right)=0.5f_{t}a_{fr}L_{fr}\frac{f_{t}}{E_{t}}\left(104F+1\right)\overset{L_{fr}=3d_{g}}{\to },$$

(31) $$w_{u,SF}=\frac{1.5f_{t}^{2}a_{fr}d_{g}\left(104F+1\right)}{f_{t,SF}E_{t}\left(k_{f}+0.5k_{w}-0.5k_{f}k_{w}\right)}$$

The stress at each stage can be calculated as:

(32) $$\sigma_{t}=\left\{\begin{array}{cc} \varepsilon_{t}E_{t,SF} & \mathrm{if}\;0<\varepsilon_{t}\leq \varepsilon_{to,SF}\\ f_{t,SF}\left(1-\frac{1-k_{f}}{k_{w}w_{u,SF}}w_{t}\right) & \mathrm{if}\;0<w_{t}\leq k_{w}w_{u,SF}\\ k_{f}f_{t,SF} & \mathrm{if}\;k_{w}w_{u,SF}<w_{t}\leq w_{u,SF} \end{array}\right\},$$

Furthermore, the following CDP-material-associated parameters define the inelastic behavior of SFRC [91] and their values used herein are presented in Table 3:

#### 2.2.3. Modeling of Steel Reinforcement

The cyclic response of the steel reinforcing bars and stirrups is derived by a superposition of several elastic and perfectly plastic models in parallel. This takes account of a nonlinear kinematic positive strain-hardening since plastic behavior is defined by the values of the yield strength and the corresponding plastic strain (*f_y_*, *ε_y_*), the ultimate strength and the corresponding strain (*f_u_*, *ε_u_*) and is characterized by permanent deformations. The values of the steel modulus of elasticity, *E_s_*, and Poisson’s ratio are also used in the FE analysis according to the test data of the steel reinforcement.

#### 2.2.4. Element Types

**Figure 6:**
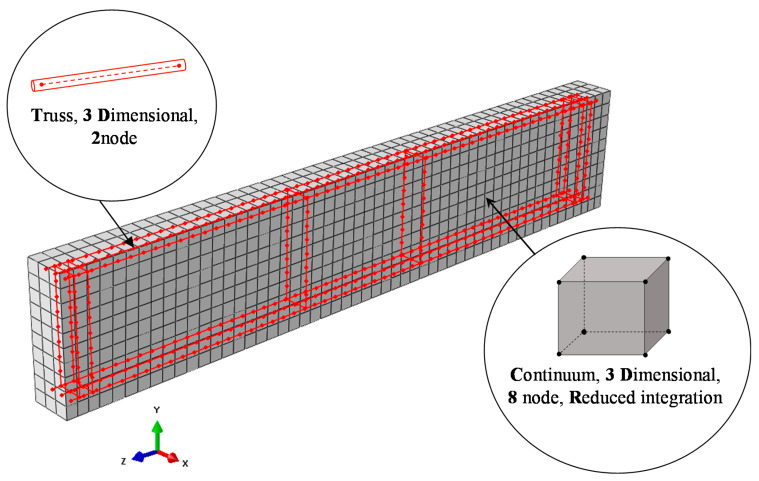
Finite element (FE) mesh of the SFRC and the steel reinforcement of the beam.

SFRC was simulated by using 8-node 3-dimensional solid elements with reduced integration (C3D8R) to avoid the effect of shear locking. The 3-dimensional 2-node truss elements (T3D2) were selected for the simulation of steel reinforcement (longitudinal and stirrups). Every element’s node has three degrees of freedom with x, y, and z (global coordinate system) translation, as depicted in Figure 6. The bond between reinforcement and concrete is modeled using the embedded process, and precisely the Abaqus feature “truss in solid” [85].

#### 2.2.5. Boundary Conditions

**Figure 7:**
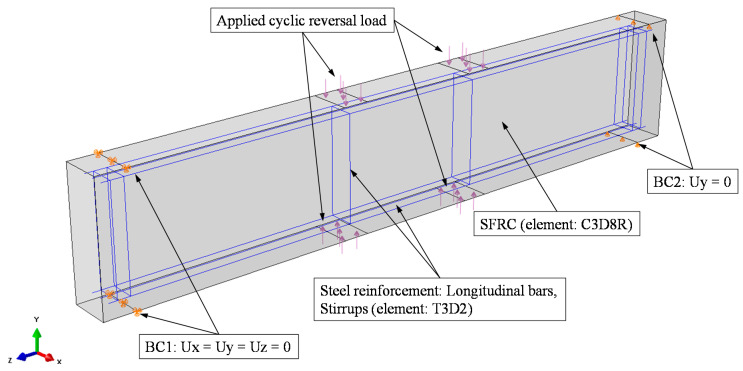
Boundary conditions and loading of the FE simulation of the beam.

The simulated beam’s boundary conditions were adopted according to the experimental setup shown in Figure 7. The supports were positioned at a particular distance from each edge, while the edges remained free. At the left side, a line of nodes was constrained in the Ux, Uy, Uz directions, while at the right side only the Uy direction was constrained.

#### 2.2.6. Loading, Mesh and Convergence

The load was applied to the beam specimens in a quasi-static manner as a reverse cyclic loading, which implies that each cycle consisted of loading in both directions, as shown in Figure 7. Analysis up to load failure capacity of the tested SFRC beams was beyond the scope of this study. The slender beams were subjected to displacement control while the deep beams to load control cyclic histories. Furthermore, the load was applied constantly and smoothly to achieve a quasi-static solution and prevent any essential acceleration alteration through each iteration, which further ensures that the stress and displacement changes remain smooth.

Mesh size was selected based on the assumption that the distribution of SFRC cracking typically includes spatial scales between two to three dominant aggregate sizes of the concrete mixture [90]. The maximum aggregate size for the slender and the deep beams was 16 and 9.5 mm, respectively. Mesh sizes of 40 and 30 mm have been applied to all types of elements (truss and solid).

#### 2.2.7. FE Simulation of the Tested Beams and Material Input

The simulations of the examined SFRC beam specimens were developed according to the geometrical and mechanical properties of the tested beams and the boundary conditions of the experimental setup. The aspects of the developed FE modeling are described in Section 2.2.1, Section 2.2.2, Section 2.2.3, Section 2.2.4, Section 2.2.5, Section 2.2.6 and Section 2.2.7 of this paper. Furthermore, the main characteristics of the materials for each specimen are presented in Table 1 and Table 2. Supplementary input parameters are presented in Table 4. Stress, *σ*, crack width, *w*, and strain, *ε*, values at points 1, 2 and 3, and the corresponding plastic damage factors, *d_t_*, of Table 4 are defined in the tensional behavioral model of the SFRC shown in Figure 5.

## 3. Results and Discussion

### 3.1. Verification of the Model

**Figure 8:**
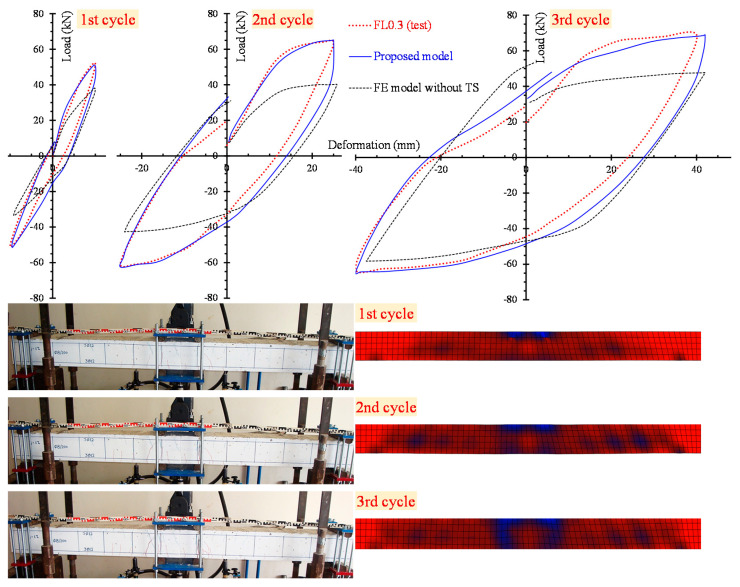
Comparisons between the analytical and experimental results of slender beam FL0.3 in load versus deformation curves and cracking patterns for the 1st, 2nd and 3rd loading cycle.

**Figure 9:**
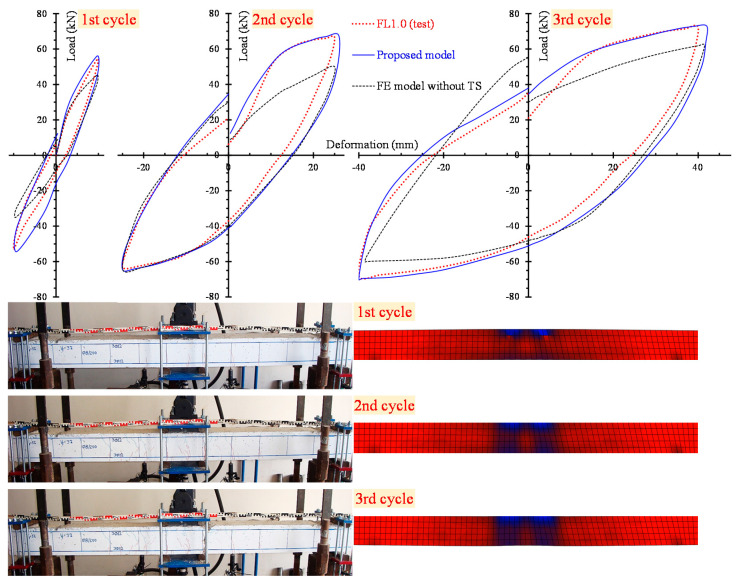
Comparisons between analytical and experimental results of slender beam FL1.0 in load versus deformation curves and cracking patterns for the 1st, 2nd and 3rd loading cycle.

The numerical results yielded from the developed nonlinear FE simulations are compared with the experimental data using load versus hysteretic deformation curves. Figure 8 and Figure 9 clearly demonstrate the analytical and the test curves of the slender beams FL0.3 and FL1.0 with steel fiber factor *F* = 0.3 (*V_SF_* = 1%) and *F* = 1.0 (*V_SF_* = 3%), respectively, for each loading cycle (see the three diagrams of the 1st, 2nd and 3rd cycle in Figure 8 and Figure 9).

In order to comprehend the effect of the tension softening and the residual stiffness on the flexural hysteretic response of the tested SFRC beams, two analytical curves are compared with the test curve in Figure 8 and Figure 9. The first curve (continuous blue line) has been derived from the proposed nonlinear FE smeared crack analysis with tension softening and residual stiffness approach and the second curve (black thin dotted line) from the FE analysis without taking into account this effect (denoted as “FE model without TS” in Figure 8 and Figure 9).

Figure 8 and Figure 9 also present the cracking patterns of the flexural beams FL0.3 and FL1.0, respectively, at each loading cycle obtained from the tests and compared to the corresponding cracking pattern at the same loading level derived from the proposed analysis using stress distribution data. Experimental and numerical crack propagation due to flexure at the end of each hysteretic loading cycle are in good compliance.

**Figure 10:**
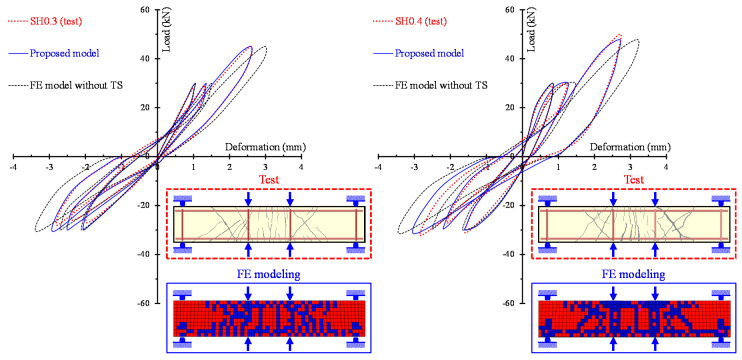
Comparisons between the analytical and experimental hysteretic response and cracking patterns of shear-critical beams without stirrups SH0.3 and SH0.4.

**Figure 11:**
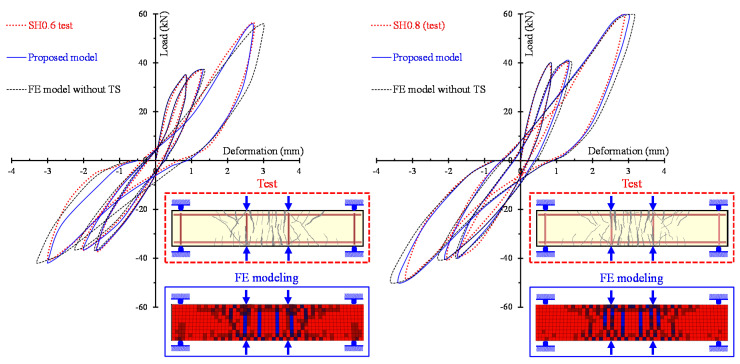
Comparisons between the analytical and experimental hysteretic response and cracking patterns of shear-critical beams without stirrups SH0.6 and SH0.8.

**Figure 12:**
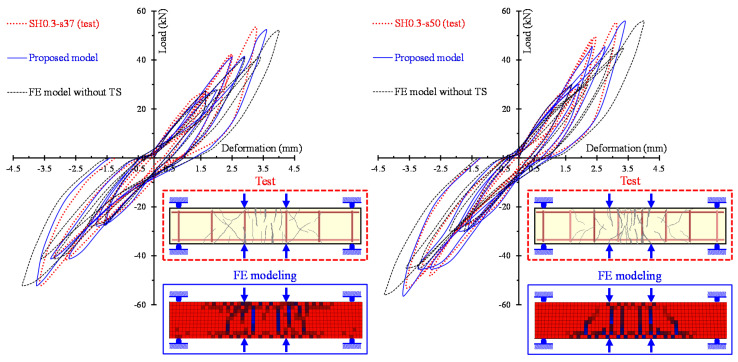
Comparisons between the analytical and experimental hysteretic response and cracking patterns of shear-critical beams with stirrups SH0.3-s37 and SH0.3-s50.

The ability of the proposed model to calculate accurately the entire hysteretic load versus the deformation behavior of SFRC beams with different failure modes and various steel fiber volumetric fractions is examined in Figure 10, Figure 11 and Figure 12. In these Figures, the analytical and the experimental hysteretic response and cracking patterns at the failure of the shear-critical beams are compared. Each load versus deformation diagram includes an experimental and two numerical curves yielded from the proposed FE analysis with tension softening and a residual stiffness effect (continuous blue line) and form the FE analysis without taking into account this effect (black thin dotted line). Figure 10 presents the diagrams of shear-critical beams without stirrups SH0.3 and SH0.4, and Figure 11 shows the diagrams of shear-critical beams SH0.6 and SH0.8, also without stirrups. Figure 12 presents the diagrams of shear-critical beams with stirrups SH0.3-s37 and SH0.3-s50.

The comparisons of the hysteretic response obtained from the tests and derived from the proposed model, as illustrated in Figure 8, Figure 9, Figure 10, Figure 11 and Figure 12, indicate that in most beams the predictions of the developed FE analysis that takes into account the smeared crack model with tension softening and residual stiffness effect are in closer agreement with the experimental data than the predictions without this effect. It is noted that numerical curves derived from the proposed model fit well to the test results in both types of beams that failed in flexure (specimens of group “FL”) and in shear (specimens of groups “SH-s” and “SH”). Furthermore, the formation of cracks during the performed cyclic tests and the corresponding cracking patterns derived from the FE model exhibit many similarities.

### 3.2. Analysis of the Hysteric Behavior and Accuracy of the Proposed Model

The load, *P*, versus displacement, *δ*, curves of the structural members under cyclic reversal loading are the basis of their hysteretic performance. Based on the test results as well as the FE analysis, the *P*-*δ* hysteretic curves for the SFRC beams have been created.

#### 3.2.1. Simplification of the hysteretic Loop

The hysteretic loop (or else full loading cycle) can be interpreted either by the actual path of the loop’s curve, or, by parameters that define its general form. One of the essential properties of the hysteretic loop is its inclination. A complete loop comprises a loading and an unloading curve. After the first crack formation on the member, the slope of the loading curve decreases with the increase in displacement, indicating that the members’ stiffness has decreased during each repeated loading cycle. Like the loading curve, the inclination of the unloading curve also declines as the number of cycles increases, and the members’ unloading stiffness gradually degrades.

**Figure 13:**
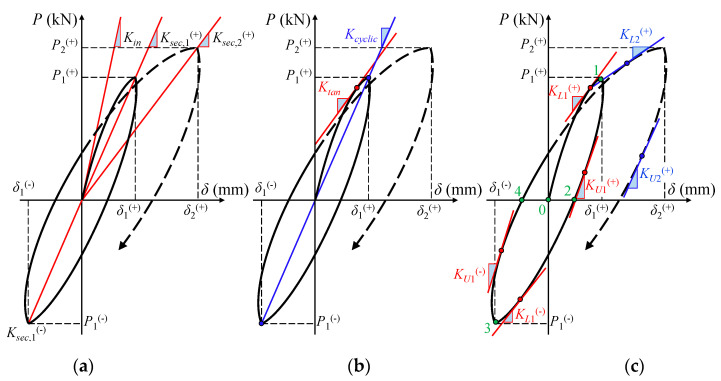
Definitions and variables of the hysteretic loop-loading cycle: (**a**) initial elastic deformation stiffness and secant stiffness; (**b**) tangent and cyclic stiffness; (**c**) loading and unloading stiffness during cyclic reversal imposed load.

When the loading of the members starts, the member’s stiffness is equal to the initial elastic deformation stiffness, *K_in_*, as shown in Figure 13a, this is also declared to be the maximum stiffness of the element. As the loading cycle proceeds, the loop inclination depends on the stiffness of the member, which can be estimated by the tangent stiffness, *K_tan_*, at any point throughout the loading phase (Figure 13b). The value of tangent stiffness varies throughout the cycle of loading, but its average value over the entire loop can be approximated by the cyclic stiffness, *K_cyclic_*. The average value of tangent stiffness, *K_tan_*, for a half loading cycle can be approximated by the secant stiffness, *K_sec_*. When the loop is symmetrical the average values of *K_sec_*^(+)^ and *K_sec_*^(-)^ equal to the value of cyclic stiffness, *K_cyclic_*. Referring to Figure 13a,b, in the linear elastic load range, *K_in_* = *K_sec_* = *K_tan_*. The use of *K_sec_* is preferred rather than *K_tan_* in the processing of test data because it is an order-of-magnitude less influenced by random errors. Nevertheless, *K_tan_* is preferred in numerical procedures that require the assembly of an incremental stiffness matrix. In this study *K_sec_*, *K_tan_* and *K_cyclic_* are calculated and compared for each cycle.

#### 3.2.2. Degradation Analysis of Strength and Stiffness

Stiffness is an important assessment index to evaluate the cyclic response of an SFRC member. Thus, stiffness at different points has been calculated and then statistically analyzed to evaluate the member’s performance and the effectiveness of the proposed model. Tangent stiffness, *K_tan_*, has been calculated at multiple points in each loading cycle, according to Figure 13b. As shown in Figure 13c, the loading starts from point 0 to the unloading point 1, and then reverse loading starts at point 2. During this process, along the path 0-1-2 the tangent stiffness of the member is changed from initial stiffness, *K_in_*, to tangent stiffness, *K_L_*_1_^(+)^, as the member’s behavior changes from elastic to post-cracking, and then again after reaching the maximum loading point 1 of unloading starts and the tangent stiffness changes again from *K_L_*_1_^(+)^ to *K_U_*_1_^(+)^. A half cycle is completed from point 0 to point 2, the reverse loading starts from point 2 and increases until the reach of maximum point 3, at which point, unloading starts again to the reverse unloading point 4. During this reverse loading process, stiffness changes again from *K_L_*_1_^(-)^ to *K_U_*_1_^(-)^. This process continuous in every next cycle until the end of the experimental testing.

#### 3.2.3. Accuracy of the Model

In order to establish the validity and check the accuracy of the proposed nonlinear FE analysis, Table 5, Table 6, Table 7 and Table 8 summarize the differences between the numerical calculations and the experimental results in terms of “calculation errors”. The discrepancy of the load, the deformation and the stiffness (tangent, secant and cyclic) between the predictions and the tests along the entire hysteretic diagrams of each beam have been calculated in order to evaluate the accuracy of the examined models. The following know expression is used to calculate the discrepancy as a percentage error:

(33) $$Error\;of\;VARiable\;\left(\%\right)=\left|\frac{VAR_{\;model}-VAR_{exp}}{V_{exp}}\right|\times 100,$$

where *VAR_model_* and *VAR_exp_* are the values of the examined variable derived from the numerical models (A: using the proposed model or B: using the FE model without tension softening and residual stiffness effect) and the experiments, respectively.

The variables examined herein are the applied load, *P*, or the deformation, *δ* (Table 5), the tangent stiffness, *K_tan_* (Table 6), the secant stiffness, *K_sec_* (Table 7) and the cyclic stiffness, *K_cyclic_* (Table 8). Each table summarizes the mean absolute error (MAE), the standard error (SE) and the coefficient of variation (CV) of the examined variable for each beam.

The overall MAE values in Table 5, Table 6, Table 7 and Table 8 indicate that the proposed model predictions (Model A) lead to an error below 5.9%, 11.2%, 8.3% and 7.3% for *P* or *δ*, *K_tan_*, *K_sec_* and *K_cyclic_*, respectively, for each tested SFRC beam and the average MAE of all tested beams is 3.3%, 9.3%, 4.8% and 4.2%, respectively, for the aforementioned variables. The corresponding average MAE of all tested beams for the same variables using the predictions of the FE model without tension softening; the stiffening effect (model B) is much higher and equal to 15.2%, 16.5%, 13.2% and 12.3%, respectively. These average values of MAE clearly indicate that the developed FE analysis that takes into account the proposed model with tension softening for the tensional behavior of SFRC and residual stiffness effect yields to accurate predictions of the hysteretic response of concrete members reinforced with conventional reinforcement (bars and stirrups) and steel fibers.

### 3.3. Effect of Steel Fibers on the Hysteretic Response

**Figure 14:**
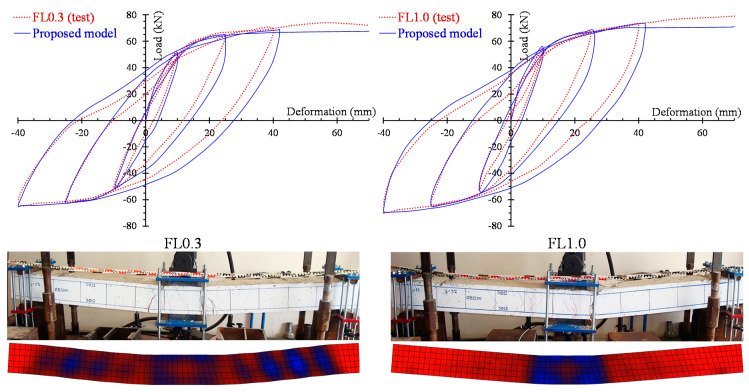
Full hysteretic response and cracking pattern at failure of the slender beams (group “FL” specimens).

The influence of steel fibers on the cyclic response of flexural and shear-critical beams is demonstrated in this section through the hysteretic response of the tested specimens in terms of load versus deformation curves and cracking patterns. The experimental and the numerical hysteretic curves and the cracking patterns of the slender beams FL0.3 and FL1.0 are presented and compared in Figure 14. It is emphasized that SFRC beam FL1.0 with a higher content of steel fibers (*F* = 1.0 and *V_SF_* = 3%) demonstrates higher strength, increased energy absorption capacity and improved cracking performance with less diagonal cracks formed at the shear spans, near the supports of the beam with regard to the SFRC beam FL0.3 with *F* = 0.3 and *V_SF_* = 1%.

**Figure 15:**
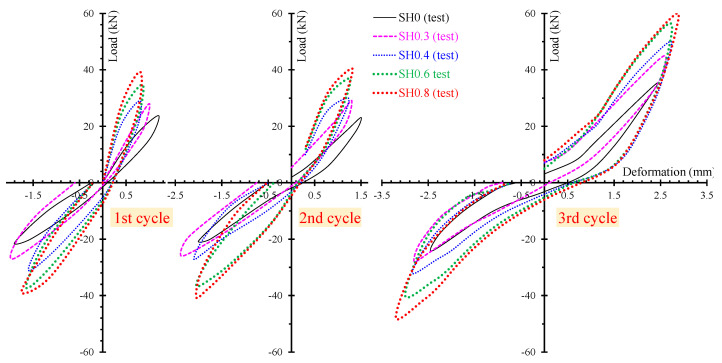
Hysteretic response at each loading cycle of the shear-critical beams without stirrups (group “SH” specimens).

The beneficial effect of the used steel fibers on the hysteretic performance is even more revealed and emphasized in the experimental results of the shear-critical beams. Figure 15 presents the load versus deformation curves per loading cycle of the beams of group “SH” (deep beams without stirrups). It is obvious that SFRC beams with a higher amount of steel fibers exhibit improved strength, stiffness and energy absorption capacity.

**Figure 16:**
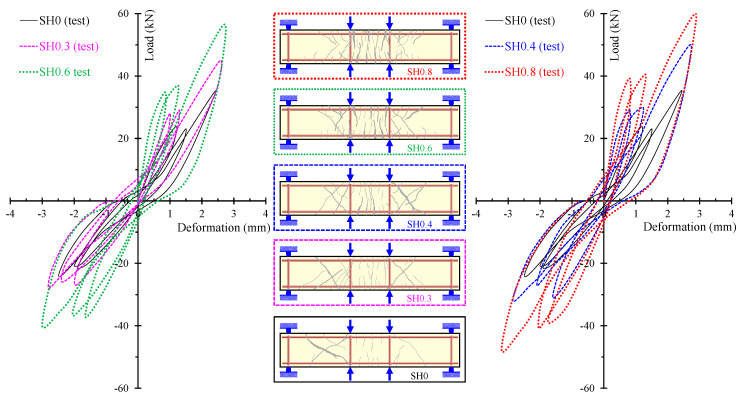
Influence of the steel fibers on the hysteretic and cracking behavior of the shear-critical beams without stirrups (group “SH” specimens).

Furthermore, the gradual increase in the amount of the steel fibers added in the SFRC beams causes a consistent enhancement of the overall hysteretic response and the cracking performance of the deep beams without stirrups, as shown in Figure 16. Furthermore, the cracking patterns shown in Figure 16 indicate that less shear diagonal cracks have been formed in the SFRC beams with a higher amount of steel fibers. In particular, SFRC specimen SH0.8 with *F* = 0.8 and *V_SF_* = 1.5% exhibited more and wide flexural cracks, whereas only slight diagonal cracks have been developed in the shear spans of the beam. On the contrary, plain concrete beam SH0 and SFRC beam SH0.3 with a low amount of steel fibers (*F* = 0.3 and *V_SF_* = 0.5%) demonstrated severe shear diagonal cracks and quite brittle behavior.

**Figure 17:**
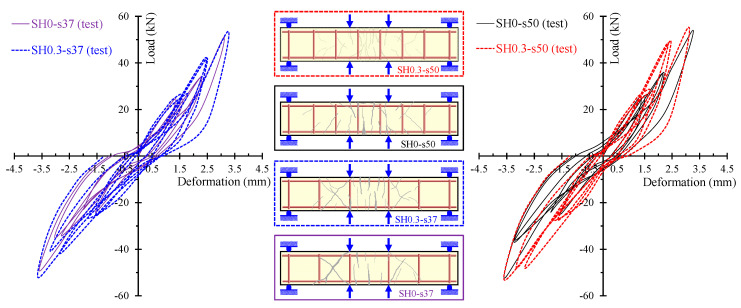
Influence of the steel fibers on the hysteretic and cracking behavior of the shear-critical beams with stirrups (group “SH-s” specimens).

More or less, similar concluding remarks are also deduced from the comparisons of the hysteretic and cracking performance of the tested deep beams with stirrups (Figure 17). Beams with steel fibers, even in low amounts (*F* = 0.3 and *V_SF_* = 0.5%) demonstrated improved overall behavior and especially cracking patterns with less severe shear diagonal cracks than the corresponding plain concrete beams.

Furthermore, the improvement of the hysteretic response due to the addition of steel fibers is also indicated in the ratios of the cumulative total energy absorbed per loading cycle of each SFRC beam summarized in Table 9. These ratios have been evaluated from the area enclosed within a full loading cycle of every cycle of the SFRC beam divided by the area of the same cycle of the corresponding reference plain concrete beam (without steel fibers). The absorbed energy reflects the capacity and toughness of the beam.

The results presented in Table 9 indicate that SFRC deep beams absorbed a substantially larger amount of energy than the beams without steel fibers since the ratios are much greater than 1.0. Although this improvement seems to be lower in the slender beams, the ability of fibers to enhance cyclic loading conditions is profound. Thus, SFRC beams maintain their integrity through a potential seismic excitation exhibiting higher energy dissipation capacities than the corresponding beams without steel fibers.

**Figure 18:**
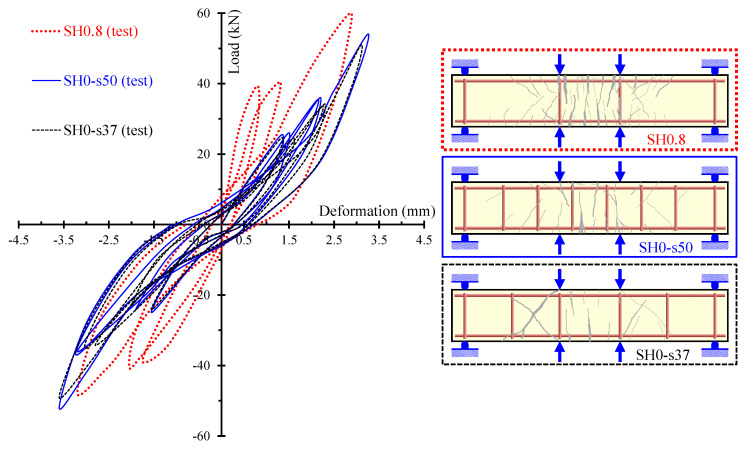
Potential replacement of common closed steel stirrups with short steel fibers.

The effectiveness of steel fibers as the only shear reinforcement, as an alternative of conventional steel stirrups is examined in Figure 18. This figure illustrates and compares the hysteretic and cracking behavior of the SFRC deep beam SH0.8 (*V_SF_* = 1.5%) without stirrups and the plain concrete deep beams SH0-s37 and SH0-s50 with stirrups ratio *ρ_w_* = 0.37% and 0.50%, respectively. From the comparison of the load versus deformation curves, it is deduced that the SFRC beam without stirrups exhibited, more or less, a comparable hysteretic response with the RC beams with stirrups. Thus, a potential replacement of stirrups with steel fibers could be achieved under certain circumstances.

These specific conditions depend on the ability of an SFRC beam with longitudinal bars to satisfy pre-set strength and ductility requirements that are defined by design criteria. The optimum amount of the steel fibers that should be added in the mixture of the beam can be evaluated using a recently proposed analytical methodology by Chalioris [92]. Based on this approach, steel fibers as the only shear reinforcement or a desirable combination of steel fibers and stirrups can be used to achieve the above requirement. The methodology is based on the fact that the desirable flexural failure mode occurs when the shear resistance of the examined SFRC beam is higher than its ultimate flexural strength. Analytical expressions to calculate the flexural and the shear strength of SFRC structural members are implemented. A formula to evaluate the minimum steel fibers required in terms of the fiber factor, *F*, has also been addressed in order for the examined SFRC beam to demonstrate pure flexural response with adequate strength and ductility, whereas its validity has been checked by the test data of 256 SFRC beams under monotonic loading from the literature [92]. However, more cyclic tests are required to provide sound conclusions concerning this important issue.

## 4. Conclusions

The efficiency of steel fibers on the hysteretic performance of realistic flexural and shear-critical steel fiber-reinforced concrete (SFRC) beams reinforced with steel reinforcements has been investigated. An experimental program of eleven beam specimens subjected to reversal cyclic loading and a numerical nonlinear finite element (FE) analysis have been presented. Based on the results of this study, the following concluding remarks can be drawn:

- The performed cyclic loading tests of slender and deep beams indicate that SFRC beams with increased values of the fiber factor, *F*, exhibit an improved hysteretic response in terms of stiffness, load-bearing capacity, deformation, energy dissipation ability and cracking behavior. The favorable effect of the used steel fibers on the overall seismic response has been highlighted since SFRC specimens maintain their integrity through the imposed reversal cyclic tests exhibiting higher values of load-bearing capacity and cumulative energy absorbed per loading cycle than the corresponding plain concrete beams without fibers. It is noted that steel fibers with a volume fraction of 1% and 3% provided a 17% and 28% increase in the energy dissipation, respectively, for the case of the flexural beams. This increase was much higher in the shear-critical beams without stirrups. In particular, the ratio of the cumulative energy absorbed of the last loading cycle of the SFRC deep beams to the corresponding energy of the reference plain concrete beams was 1.60, 2.40, 3.09 and 3.47 for beams with fiber factor *F* = 0.3, 0.4, 0.6 and 0.8, respectively.
- Shear-critical beams reinforced with longitudinal bars and steel fibers without stirrups exhibited comparable hysteretic response in terms of strength and absorbed energy with the corresponding deep beams reinforced with bars and stirrups without steel fibers. Although more tests are still required to provide wide-ranging conclusions, it is indicated that a potential replacement of stirrups with steel fibers could be achieved under certain circumstances that depend on the ability of the SFRC beam to satisfy pre-set strength and ductility requirements.
- The developed FE simulation considers the nonlinearities of the materials by a smeared crack approach with tension softening and residual stiffness effect. The favorable influence of the steel fibers is evaluated according to their characteristics and content in order to achieve a more realistic prediction of SFRC behavior under compression and tension.
- The direct tension experimental results of SFRC specimens carried out in this study verify the analytical predictions of the proposed tensional model. These tests indicate that steel fibers substantially improve the post-cracking tensile behavior and the residual stress versus crack width curve according to the values of the fiber factor, *F*. Specifically, the value of the maximum post-cracking residual tensile stress was found to be 0.22, 0.33, 0.40 and 0.57 times the value of the tensile strength for SFRC mixtures with fiber factor *F* = 0.3, 0.4, 0.6 and 0.8, respectively. Furthermore, this fiber factor is a more efficient parameter than the volume fraction for the evaluation of the steel fiber contribution.
- The developed nonlinear FE analysis accurately predicts the overall hysteretic response and points out the beneficial effect of the added fibers. Comparisons between the test and numerical results reveal that the developed nonlinear FE analysis with a smeared crack model that takes into account the tension softening and residual stiffness effect accurately predicts the hysteretic response of realistic SFRC beams with steel reinforcement. Furthermore, its validity and accuracy have been checked by calculating the discrepancies between test data and numerical predictions for various variables, such as load, deformation, and stiffness. The mean absolute errors of these variables were found to be satisfactorily low for the tested beams.

## Figures and Tables

**Table 1:** Properties of the tested beams.

Group	Beam Name	Geometrical Data					Steel Reinforcement (Bars and Stirrups)					Steel Fiber Characteristics *		
		*b* (mm)	*h* (mm)	*d* (mm)	*a_s_* (mm)	*a_s/_d*	*A_s_*_1_ = *A_s_*_2_ (Ø in mm)	*ρ_l1_* = *ρ_l_*_2_ (%)	Ø*_w_*/*s* (mm/mm)	*ρ_w_* (%)	*f_y_* (MPa)	*V_SF_* (%)	*l_SF_*/*d_SF_* (mm/mm)	*F*
“FL”	FL0.3	200	200	170	1000	5.9	3Ø12	1.00	Ø8/200	0.25	590	1.00	44/1.0	0.3
	FL1.0	200	200	170	1000	5.9	3Ø12	1.00	Ø8/200	0.25	590	3.00	44/1.0	1.0
“SH-s”	SH0-s37	100	300	275	550	2.0	3Ø8	0.55	Ø8/275	0.37	575	–	–	–
	SH0-s50	100	300	275	550	2.0	3Ø8	0.55	Ø8/200	0.50	575	–	–	–
	SH0.3-s37	100	300	275	550	2.0	3Ø8	0.55	Ø8/275	0.37	575	0.50	60/0.8	0.3
	SH0.3-s50	100	300	275	550	2.0	3Ø8	0.55	Ø8/200	0.50	575	0.50	60/0.8	0.3
“SH”	SH0	100	300	275	550	2.0	3Ø8	0.55	–	–	575	–	–	–
	SH0.3	100	300	275	550	2.0	3Ø8	0.55	–	–	575	0.50	60/0.8	0.3
	SH0.4	100	300	275	550	2.0	3Ø8	0.55	–	–	575	0.75	60/0.8	0.4
	SH0.6	100	300	275	550	2.0	3Ø8	0.55	–	–	575	1.00	60/0.8	0.6
	SH0.8	100	300	275	550	2.0	3Ø8	0.55	–	–	575	1.50	60/0.8	0.8

* Hooked steel fibers with *f_SF_* = 1000 MPa.

**Table 2:** Compressive and tensile properties of the steel fiber-reinforced concrete (SFRC) of each tested beam.

Group	Beam Name	*Ε_c_* = *E_t,SF_* (GPa)	*f_c,SF_* (MPa)	*ε_cu,SF_* (mm/m)	*f_t,SF_* (MPa)	*k_f_*	*G_f,SF_* (N/mm)	*ε_to,SF_* (mm/m)	*k_w_*
“FL”	FL0.3	29.336	25.51	3.59	3.30	0.19	2.001	0.113	0.01
	FL1.0	30.442	27.25	8.47	4.39	0.43	8.561	0.144	0.01
“SH-s”	SH0.3-s37	30.055	28.76	3.43	2.39	0.22	0.655	0.080	0.04
	SH0.3-s50	30.055	28.76	3.43	2.39	0.22	0.655	0.080	0.04
“SH”	SH0.3	30.055	28.76	3.43	2.39	0.22	0.601	0.080	0.04
	SH0.4	30.264	29.64	4.33	2.44	0.33	0.892	0.081	0.04
	SH0.6	30.474	30.52	5.33	2.69	0.40	1.397	0.088	0.04
	SH0.8	30.893	32.27	7.64	2.79	0.57	2.084	0.090	0.04

**Table 3:** Concrete damaged plasticity (CDP) model input parameters.

Parameter	Value
*ψ*	40°
*K_c_*	2/3
*σ_b0_/σ_c0_*	1.16
∈	0.10
*μ*	0.0001

Where *ψ* is the dilatation angle affecting the plastic deformation, ∈ is the flow potential eccentricity that defines the rate of the plastic potential hyperbolic to its asymptote, *σ_bo_/σ_co_* is the ratio of the strength in the biaxial state to the strength in the uniaxial state, *K_c_* is the ratio of the tensile to the compressive meridian and *μ* is the viscosity parameter.

**Table 4:** Supplementary input variables for the simulation of the tested SFRC beams.

Beam Name	*ν*	*σ_t_*_1_ (MPa)	*w_t_*_1_ (mm)	*ε_t_*_1_ (mm/m)	*σ_t_*_2_ (MPa)	*w_t_*_2_ (mm)	*ε_t_*_2_ (mm/m)	*σ_t_*_3_ (MPa)	*w_t_*_3_ (mm)	*ε_t_*_3_ (mm/m)	*d_t_*_1_	*d_t_*_2_	*d_t_*_3_
FL0.3	0.212	2.56	0.012	0.09	1.07	0.034	0.26	0.62	0.045	0.34	0.225	0.676	0.811
FL1.0	0.237	3.69	0.013	0.10	2.29	0.041	0.31	1.87	0.053	0.40	0.159	0.478	0.573
SH0.3-s37	0.210	1.87	0.011	0.06	0.84	0.032	0.18	0.53	0.045	0.25	0.219	0.656	0.787
SH0.3-s50	0.210	1.87	0.011	0.06	0.84	0.032	0.18	0.53	0.045	0.25	0.219	0.656	0.787
SH0.3	0.210	1.87	0.011	0.06	0.84	0.032	0.18	0.53	0.043	0.24	0.216	0.648	0.778
SH0.4	0.216	1.98	0.011	0.06	1.07	0.032	0.18	0.80	0.041	0.23	0.187	0.561	0.673
SH0.6	0.215	2.24	0.011	0.06	1.33	0.034	0.19	1.06	0.045	0.25	0.168	0.504	0.605
SH0.8	0.231	2.46	0.011	0.06	1.79	0.032	0.18	1.59	0.043	0.24	0.119	0.357	0.428

**Table 5:** Error calculation in the prediction of load, *P* (slender beams), or deformation, *δ* (deep beams).

Beam Name		MAE at Each Cycle					Overall MAE	SE	CV
		Cycle 1	Cycle 2	Cycle 3	Cycle 4	Cycle 5			
FL0.3	A	1.6%	1.8%	1.7%	–	–	1.7%	0.4%	26%
	B	30.3%	33.8%	19.2%	–	–	27.8%	4.3%	16%
FL1.0	A	2.6%	1.9%	2.2%	–	–	2.2%	0.3%	12%
	B	25.4%	13.2%	12.7%	–	–	17.1%	4.6%	27%
SH0.3-s37	A	1.6%	9.5%	3.9%	7.7%	6.8%	5.9%	1.7%	29%
	B	3.2%	4.2%	17.6%	25.7%	19.1%	13.9%	3.7%	26%
SH0.3-s50	A	0.1%	5.6%	1.1%	8.1%	6.2%	4.2%	1.5%	36%
	B	3.1%	22.2%	22.4%	26.9%	24.2%	19.8%	3.7%	19%
SH0.3	A	5.1%	5.1%	1.8%	–	–	4.0%	0.9%	23%
	B	6.3%	15.1%	17.5%	–	–	13.0%	2.2%	17%
SH0.4	A	3.9%	3.6%	2.6%	–	–	3.4%	1.0%	27%
	B	4.7%	18.9%	18.9%	–	–	14.2%	3.0%	20%
SH0.6	A	0.1%	3.2%	0.0%	–	–	1.1%	0.7%	64%
	B	1.6%	8.9%	10.6%	–	–	7.0%	1.8%	26%
SH0.8	A	3.2%	3.1%	5.8%	–	–	4.0%	1.0%	24%
	B	4.6%	9.9%	11.5%	–	–	8.7%	1.5%	18%

A: Proposed model with tension softening and residual stiffness effect. B: FE model without TS (without tension softening and residual stiffness effect).

**Table 6:** Error calculation in the prediction of tangent stiffness, *K_tan_*.

Beam Name		MAE at Each Cycle										Overall MAE	SE	CV
		Cycle 1		Cycle 2		Cycle 3		Cycle 4		Cycle 5				
		*K_tan_* ^(+)^	*K_tan_* ^(-)^	*K_tan_* ^(+)^	*K_tan_* ^(-)^	*K_tan_* ^(+)^	*K_tan_* ^(-)^	*K_tan_* ^(+)^	*K_tan_* ^(-)^	*K_tan_* ^(+)^	*K_tan_* ^(-)^			
FL0.3	A	1.8%	3.5%	8.3%	14.2%	6.0%	6.1%	–	–	–	–	6.7%	1.6%	24.3%
	B	31.8%	41.5%	30.5%	28.4%	35.8%	24.5%	–	–	–	–	32.1%	2.0%	6.1%
FL1.0	A	5.9%	4.4%	21.6%	13.3%	4.1%	11.7%	–	–	–	–	10.2%	2.4%	24.0%
	B	25.6%	30.9%	17.4%	13.2%	16.1%	19.0%	–	–	–	–	20.4%	2.5%	12.5%
SH0.3-s37	A	5.4%	6.4%	3.2%	12.5%	1.1%	8.0%	9.2%	6.6%	6.6%	8.3%	6.7%	1.3%	18.8%
	B	5.7%	8.8%	4.7%	12.0%	16.8%	6.7%	23.0%	14.0%	23.8%	20.6%	13.6%	1.7%	12.6%
SH0.3-s50	A	8.8%	11.5%	4.8%	11.7%	9.3%	5.1%	9.4%	6.2%	13.3%	1.3%	8.1%	1.0%	12.6%
	B	8.0%	8.0%	7.2%	10.4%	23.0%	18.6%	26.7%	16.7%	23.4%	19.9%	16.2%	1.9%	12.0%
SH0.3	A	4.5%	9.3%	7.2%	23.1%	10.8%	6.1%	–	–	–	–	10.1%	2.9%	28.7%
	B	3.5%	7.4%	9.2%	14.1%	3.8%	9.9%	–	–	–	–	8.0%	2.1%	26.2%
SH0.4	A	8.0%	5.4%	14.2%	6.2%	7.5%	24.3%	–	–	–	–	10.9%	2.5%	22.5%
	B	8.0%	6.1%	22.7%	9.8%	20.2%	30.3%	–	–	–	–	16.2%	3.1%	19.2%
SH0.6	A	14.8%	10.4%	17.6%	7.0%	6.5%	8.4%	–	–	–	–	10.8%	1.5%	14.1%
	B	13.4%	10.4%	23.0%	11.1%	12.4%	21.5%	–	–	–	–	15.3%	2.0%	13.1%
SH0.8	A	12.9%	7.4%	11.0%	18.2%	10.4%	6.9%	–	–	–	–	11.2%	2.3%	20.4%
	B	12.9%	9.2%	16.2%	6.2%	11.7%	7.2%	–	–	–	–	10.6%	2.2%	20.9%

A: Proposed model with tension softening and residual stiffness effect. B: FE model without TS (without tension softening and residual stiffness effect).

**Table 7:** Error calculation in the prediction of secant stiffness, *K_sec_*.

Beam Name		MAE at Each Cycle					Overall MAE	SE	CV
		Cycle 1	Cycle 2	Cycle 3	Cycle 4	Cycle 5			
FL0.3	A	4.7%	1.8%	4.0%	–	–	3.5%	1.0%	28.2%
	B	25.9%	36.1%	29.7%	–	–	30.6%	1.9%	6.2%
FL1.0	A	8.3%	12.5%	4.1%	–	–	8.3%	1.8%	21.5%
	B	22.1%	1.9%	12.0%	–	–	12.0%	4.3%	35.9%
SH0.3-s37	A	4.8%	0.4%	4.0%	8.1%	11.1%	5.7%	1.7%	30.1%
	B	5.8%	8.0%	15.9%	19.4%	20.5%	13.9%	2.8%	19.9%
SH0.3-s50	A	7.4%	1.9%	6.2%	12.8%	10.0%	7.7%	1.7%	21.8%
	B	4.0%	12.0%	23.0%	26.0%	27.9%	18.6%	3.4%	18.3%
SH0.3	A	3.9%	4.4%	2.8%	–	–	3.7%	1.4%	37.6%
	B	2.6%	5.4%	11.0%	–	–	6.3%	2.1%	33.4%
SH0.4	A	2.9%	3.0%	5.4%	–	–	3.8%	0.7%	19.0%
	B	4.4%	11.4%	18.4%	–	–	11.4%	2.8%	24.3%
SH0.6	A	0.9%	2.9%	2.9%	–	–	2.3%	0.7%	30.7%
	B	2.3%	7.7%	7.7%	–	–	5.9%	1.5%	25.1%
SH0.8	A	3.3%	3.0%	3.5%	–	–	3.3%	0.5%	15.3%
	B	2.2%	9.0%	8.5%	–	–	6.6%	1.6%	23.8%

A: Proposed model with tension softening and residual stiffness effect. B: FE model without TS (without tension softening and residual stiffness effect).

**Table 8:** Error calculation in the prediction of cyclic stiffness, *K_cyclic_*.

Beam Name		MAE at Each Cycle					Overall MAE	SE	CV
		Cycle 1	Cycle 2	Cycle 3	Cycle 4	Cycle 5			
FL0.3	A	4.6%	1.8%	1.0%	–	–	2.5%	1.1%	44.2%
	B	25.8%	33.7%	19.2%	–	–	26.2%	4.2%	15.9%
FL1.0	A	8.2%	12.4%	0.3%	–	–	7.0%	3.5%	50.6%
	B	22.0%	3.0%	11.2%	–	–	12.1%	5.5%	45.4%
SH0.3-s37	A	3.7%	7.5%	0.0%	12.1%	7.3%	6.1%	2.0%	33.1%
	B	2.1%	2.8%	15.4%	22.9%	22.5%	13.1%	4.6%	34.8%
SH0.3-s50	A	7.0%	1.9%	5.2%	12.5%	9.9%	7.3%	1.8%	25.2%
	B	3.9%	12.0%	22.8%	25.8%	23.7%	17.7%	4.2%	23.7%
SH0.3	A	3.8%	3.4%	1.7%	–	–	3.0%	0.7%	22.0%
	B	2.2%	5.2%	11.9%	–	–	6.4%	2.9%	44.9%
SH0.4	A	2.0%	6.3%	5.8%	–	–	4.7%	1.3%	28.5%
	B	4.0%	7.1%	18.7%	–	–	9.9%	4.5%	44.9%
SH0.6	A	0.4%	0.5%	1.6%	–	–	0.8%	0.4%	45.4%
	B	2.3%	7.9%	8.1%	–	–	6.1%	1.9%	31.1%
SH0.8	A	0.0%	2.9%	3.7%	–	–	2.2%	1.1%	50.2%
	B	1.7%	9.5%	8.7%	–	–	6.6%	2.5%	37.4%

A: Proposed model with tension softening and residual stiffness effect. B: FE model without TS (without tension softening and residual stiffness effect).

**Table 9:** Energy dissipation ratios.

Group	Beam Name	*F*	*V_SF_* (%)	*ρ_w_* (%)	Cycle 1	Cycle 2	Cycle 3	Cycle 4	Cycle 5
“FL”	FL0.3	0.3	1.00	0.25	1.00	1.17	1.09	–	–
	FL1.0	1.0	3.00	0.25	1.00	1.28	1.20	–	–
“SH-s”	SH0.3-s37	0.3	0.50	0.37	1.33	1.80	1.59	1.26	1.49
	SH0.3-s50	0.3	0.50	0.50	1.42	1.73	1.40	1.21	1.27
“SH”	SH0.3	0.3	0.50	–	1.38	1.60	1.60	–	–
	SH0.4	0.4	0.75	–	1.40	1.67	2.40	–	-
	SH0.6	0.6	1.00	–	1.73	2.08	3.09	–	–
	SH0.8	0.8	1.50	–	1.98	2.55	3.47	–	–
